# Amniotic Fluid microRNA in Severe Twin-Twin Transfusion Syndrome Cardiomyopathy—Identification of Differences and Predicting Demise

**DOI:** 10.3390/jcdd9020037

**Published:** 2022-01-23

**Authors:** Eleanor L. Schuchardt, Shelley D. Miyamoto, Timothy Crombleholme, Anis Karimpour-Fard, Armin Korst, Bonnie Neltner, Lisa W. Howley, Bettina Cuneo, Carmen C. Sucharov

**Affiliations:** 1 Department of Pediatrics, Colorado Fetal Care Center, Children’s Hospital Colorado, School of Medicine, University of Colorado, Aurora, CO 80045, USA; ESchuchardt@health.ucsd.edu (E.L.S.); shelley.miyamoto@childrenscolorado.org (S.D.M.); Bettina.Cuneo@childrenscolorado.org (B.C.); 2 Department of Pediatrics, Rady Children’s Hospital, School of Medicine, University of California San Diego, San Diego, CA 92123, USA; 3 Fetal Care Center Dallas, Medical City Children’s Hospital, Dallas, TX 75230, USA; timothy.crombleholme@fetalcaredallas.com; 4 Department of Pharmacology, School of Medicine, Anschutz Medical Campus, University of Colorado, Aurora, CO 80045, USA; anis.karimpour-fard@cuanschutz.edu; 5 Research Institute, Children’s Hospital Colorado, Aurora, CO 80045, USA; armin.korst@childrenscolorado.org; 6 Division of Cardiology, Department of Medicine, University of Colorado School of Medicine, Aurora, CO 80045, USA; bonnie.neltner@cuanschutz.edu; 7 Division of Cardiology, Department of Pediatrics, The Children’s Heart Clinic, Children’s Minnesota, Minneapolis, MN 55404, USA; lhowley@chc-pa.org

**Keywords:** micro-RNA, twin-twin transfusion syndrome, fetal cardiomyopathy, biomarker

## Abstract

Twin-twin transfusion syndrome (TTTS) is a rare but serious cause of fetal cardiomyopathy with poorly understood pathophysiology and challenging prognostication. This study sought a nonbiased, comprehensive assessment of amniotic fluid (AF) microRNAs from TTTS pregnancies and associations of these miRNAs with clinical characteristics. For the discovery cohort, AF from ten fetuses with severe TTTS cardiomyopathy were selected and compared to ten normal singleton AF. Array panels assessing 384 microRNAs were performed on the discovery cohort and controls. Using a stringent *q* < 0.0025, arrays identified 32 miRNAs with differential expression. Top three microRNAs were miR-99b, miR-370 and miR-375. Forty distinct TTTS subjects were selected for a validation cohort. RT-PCR targeted six differentially-expressed microRNAs in the discovery and validation cohorts. Expression differences by array were confirmed by RT-PCR with high fidelity. The ability of these miRNAs to predict clinical differences, such as cardiac findings and later demise, was evaluated on TTTS subjects. Down-regulation of miRNA-127-3p, miRNA-375-3p and miRNA-886 were associated with demise. Our results indicate AF microRNAs have potential as a diagnostic and prognostic biomarker in TTTS. The top microRNAs have previously demonstrated roles in angiogenesis, cardiomyocyte stress response and hypertrophy. Further studies of the mechanism of actions and potential targets is warranted.

## 1. Introduction

Twin-twin transfusion syndrome (TTTS) is a rare but serious condition which affects 10–15% of monochorionic twin pregnancies and is a major cause of fetal cardiomyopathy. It accounts for 15% of all twin perinatal mortality and is the most frequent cause of fetal death in monochorionic pregnancies [1,2]. In the era of fetal intervention, high volume centers achieve up to 70% survival, though timely diagnosis and intervention remain key [3]. Survivors of TTTS often face complications due to prematurity, growth aberrations, neurologic injury and cardiovascular disease [2]. 

Knowledge of TTTS pathophysiology is limited. It is postulated that placental vascular connections develop between twins during the mid-trimester phase of rapid placental growth and facilitate an imbalance of fluid and vasoactive mediators between twins. This results in a hypovolemic state in the donor twin and a fluid- and pressure-overloaded state in the recipient twin, which progresses to a hypertensive cardiomyopathy. The fluid overload also results in natriuretic peptide production, which manifests as polyhydramnios. In contrast, the donor twin experiences hypovolemia, leading to activation of the renin-angiotensin-aldosterone system (RAAS) and resultant vasoconstriction and oligohydramnios. The placental anastomoses allow for transfer of RAAS components from the donor to the recipient twin and thus create a vicious cycle [4]. Essentially, TTTS is a placental disease with significant cardiac ramifications. 

The diagnosis and staging of TTTS is made by obstetric ultrasound. Multiple systems of severity classification have been developed; first, the Quintero staging system and later the Cincinnati modification and the Children’s Hospital of Philadelphia (CHOP) score, which incorporate cardiac findings into the diagnosis [3,5,6]. The Tei index (or myocardial performance index) is frequently used as a quantitative marker of systolic and diastolic function in TTTS in which higher values indicate more dysfunction [5]. However, these systems all fail to predict disease progression or fetal demise [3]. Because of these diagnostic and prognostic challenges, there have been several prior investigations to assess potential biomarkers for TTTS, both in amniotic fluid and maternal serum [7,8,9,10]. A TTTS biomarker could focus the surveillance of monochorionic pregnancies, assist in earlier detection of disease progression or, potentially, identify those at highest risk of demise.

microRNAs (miRNAs) are short, noncoding RNAs 20–22 nucleotides in length that exert regulatory effects on messenger RNAs and, subsequently, impact gene expression [11]. They are found in a wide variety of bodily fluids and tissues and are remarkably stable in fluids despite the presence of RNases [11,12]. Circulating and tissue specific miRNAs have shown to be useful biomarkers for heart failure, cardiomyopathy, hypertension, coronary disease and arrhythmias [12,13,14,15,16,17]. In addition, due to their silencing effect on gene expression, miRNAs pose unique potential as a future therapeutic target [18]. 

Amniotic fluid miRNAs are of emerging interest in several fetal pathologies [11,19,20]. Amniotic fluid can be easily sampled during selective fetoscopic laser photocoagulation (SFLP), which is the definitive therapy for TTTS and interrupts the placental vascular connections between twins [2]. Our group has previously found AF miRNAs differences in a small comparison of TTTS subjects with (*n* = 14) and without (*n* = 12) severe cardiomyopathy [21]. A more comprehensive comparison to normal pregnancies has not been performed to date. We sought to perform that comparison and utilize those differences to identify predictors of fetal outcome.

We hypothesized amniotic fluid miRNAs would differentiate TTTS from singleton pregnancies with many differences. The purpose of this study was to perform a non-biased, comprehensive assessment of the miRNA profile in amniotic fluid from TTTS pregnancies to further establish amniotic fluid miRNAs as a potential diagnostic or prognostic biomarker of TTTS. Furthermore, we sought to evaluate if specific differentially expressed miRNAs were associated with fetal growth restriction, more severe cardiac findings, or fetal demise in a larger population of TTTS fetuses. Additionally, we sought to demonstrate fidelity between array and RT-PCR methodologies.

## 2. Materials and Methods

### 2.1. Subject Selection

TTTS: Amniotic fluid from fetuses undergoing fetoscopic intervention was banked from January 2015–May 2018 at a single fetal center (Colorado Fetal Care Center, Aurora, CO, USA). Ten subjects—those with the most severe disease based on Quintero stage and RV Tei index—were selected for the discovery cohort (Table 1). Forty distinct additional TTTS subjects were selected for the validation cohort. These were matched for gestational age with the discovery cohort. Subjects were excluded if structural heart disease was present at time of diagnosis. Amniotic fluid was obtained upon entry to the recipient twin’s amniotic sac prior to SFLP. Extracted fluid was immediately placed on ice and spun at 4 °C. The supernatant was frozen at −80 °C. 

Controls: Amniotic fluid from singleton pregnancies previously obtained for clinical karyotyping at the Colorado Genetics Laboratory (CGL) was used for control comparison (Table 1). Anonymized samples were matched for fetal sex and gestational age. Exclusion criteria for control subjects included perinatal infection, congenital heart disease and major extracardiac anomalies such as abdominal wall defect, congenital diaphragmatic hernia, bladder outlet obstruction or tracheoesophageal fistula. Control samples were stored at room temperature before arriving to CGL, then spun, aliquoted and stored at −20 °C.

### 2.2. miRNA Extraction

Total RNA was extracted from 300 μL amniotic fluid using the miRNeasy Mini Kit (Qiagen, Venlo, Limburg, The Netherlands) per manufacturer’s recommendations. 

### 2.3. miRNA Array

Arrays were performed utilizing 3 μL of total RNA. RNAs were reverse transcribed using a primer pool specific for each miRNA. These products were subsequently pre-amplified using a specific primer pool. Pre-amplification products were loaded on a 384-well plate-A TaqMan Low Density Array (Applied Biosystems TaqMan MicroRNA Arrays, ThermoFisher Scientific, Waltham, MA, USA) and amplified in the ABI7900HT. Although the number of targets identified in the TaqMan Low Density array is lower than a traditional array, our group has standardized the conditions to identify circulating miRNAs using this platform. Detailed method description was previously described by our group [20].

### 2.4. Array Analysis

Array data were analyzed with the Expression Suite Software (version 1.1 ThermoFisher Scientific). Internal controls were determined based on an algorithm generated by the software and confirmed by cycle threshold (Ct) analysis to identify the miRNAs with the least variability between samples. Both methods independently identified miR-363 as the least variable miRNA. 

After normalization, classification of miRNAs was performed using R (www.R-project.org, accessed on 25 November 2019). Heat maps were generated based on *t*-test Wilcoxon of miRNAs with a q-value < 0.0025 using a function in R. Random Forest (RF) analysis was used to identify the top 3 miRNAs that differentiated TTTS from singleton controls. Area under the receiver operating characteristic curve (AUC) was calculated in R. ROC curve were generated to determine the sensitivity and specificity of the top 3 differentiating miRNAs [22].

### 2.5. Degradation Experiment

Due to differing handling conditions of the TTTS subjects’ and control subjects’ amniotic fluid, an additional experiment assessing the degradation of miRNAs in amniotic fluid was designed and performed. Prospectively, two patients with TTTS undergoing SFLP who consented to participate were selected. Amniotic fluid from these patients was sampled per usual procedure. Fluid was aliquoted and exposed to 3 conditions: (1) immediate placement on ice, spun and frozen to −80 °C per usual procedure for TTTS samples, (2) maintained at room temperature for 4 hours before being spun and frozen to −20 °C, (3) maintained at room temperature for 24 h before being spun and frozen to −20 °C. From these 6 aliquots, RNA extraction, miRNA arrays and data analysis were performed per procedure described elsewhere in Methods. These subjects were not included in the overall array analysis.

### 2.6. miRNA RT-PCR and Analysis

To validate array results, miRNA expression was evaluated by reverse transcriptase-polymerase chain reaction (RT-PCR). RT-PCR was performed on all TTTS subjects in the discovery cohort, seven control subjects and the 40 TTTS subjects of the validation cohort. miRNA extraction was performed using the methods detailed above. miRNAs for RT-PCR were selected based on q-value and RF analysis and included miR-370, miR-375, miR-886-5p, miR-127, miR-99b. Reverse transcription (RT) of these select miRNAs was performed with the use of the miScript Reverse Transcription Kit (Qiagen, ThermoFisher Scientific, Waltham, MA, USA ) according to manufacturers’ recommendations. Taqman primer kits for each of the selected miRNAs were purchased from ThermoFisher Scientific. miR-363 was used as normalizer.

Statistical analyses were performed using GraphPad Prism (GraphPad Software) (version 9.3.0, San Diego, CA, USA 92108). Significance threshold was set a priori at *p* < 0.05, and all data were checked for normality using Shapiro-Wilk. Quantitative results are presented as mean ± SEM. Unpaired *t*-test was used for comparisons between 2 normally distributed groups.

### 2.7. Association between Clinical Characteristics and miRNA Levels

To evaluate if specific highly-differentially expressed miRNAs were associated with clinical characteristics or outcome, we divided our TTTS subject group (*n* = 50) and compared miR-370, miR-375, miR-886-5p, miR-127, miR-99b levels between groups. Comparisons included TTTS subjects (1) with and without growth restriction, (2) with and without a fetal demise, (3) with moderate or severe tricuspid regurgitation to those with mild or less, (4) with moderate or severe ventricular hypertrophy to those without. Group comparisons were performed by two-tailed *t*-test and simple linear regression analysis were performed using GraphPad Prism (version 9.3.0, San Diego, CA, USA).

### 2.8. Pathway Analysis

To identify putative pathways or targets of miRNAs, KEGG pathway analysis was done using the miRNA Enrichment Analysis and Annotation Tool (miEAA, version 2.0). Pathway analyses were performed on the 32 differentially expressed miRNAs by *q* < 0.0025 and separately on the miRNAs investigated by RT-PCR (miR-370, miR-375, miR-886-5p, miR-127, miR-99b). A *p*-value < 0.001 defined significance.

## 3. Results

### 3.1. Subject Characteristics

Amniotic fluid from 60 fetuses were analyzed (Table 1). The median and interquartile gestational age range of the controls, TTTS discovery cohort and TTTS validation cohort were 19.43 (17.21–22.18), 19.71 (17.64–22.39) and 19.71 (18–21.18) weeks, respectively (*p*-value = 0.88). 

Among the discovery TTTS cohort, all subjects had Cincinnati Stage 3C TTTS with recipient twin RV and LV Tei indices prior to SFLP of 0.80 (0.71–0.9) and 0.65 (0.58–0.67), respectively (median, IQR). There were no recipient twin demises and three donor demises after SFLP. Six donors had severe fetal growth restriction (defined as estimated fetal weight < 10% for gestational age). Three recipients had moderate or more tricuspid regurgitation. 

Among the validation TTTS cohort, 39 had Cincinnati Stage 3C TTTS and one had Stage 2B. The recipient twin RV and LV Tei indices prior to SFLP were 0.65 (0.59–0.69) and 0.6 (0.53–0.68), respectively. One twin pair experienced a double demise, seven experienced donor twin demise and one experienced isolated recipient demise. All demises occurred after SFLP. The timing or etiology of demise was not always known; however available information is reported in Supplemental Table S1. Fetal growth restriction was common, affecting 28 fetuses. Eleven recipients had moderate or more tricuspid regurgitation. 

### 3.2. Array Results: miRNAs Differentiate TTTS and Singleton Controls

**Figure 1:**
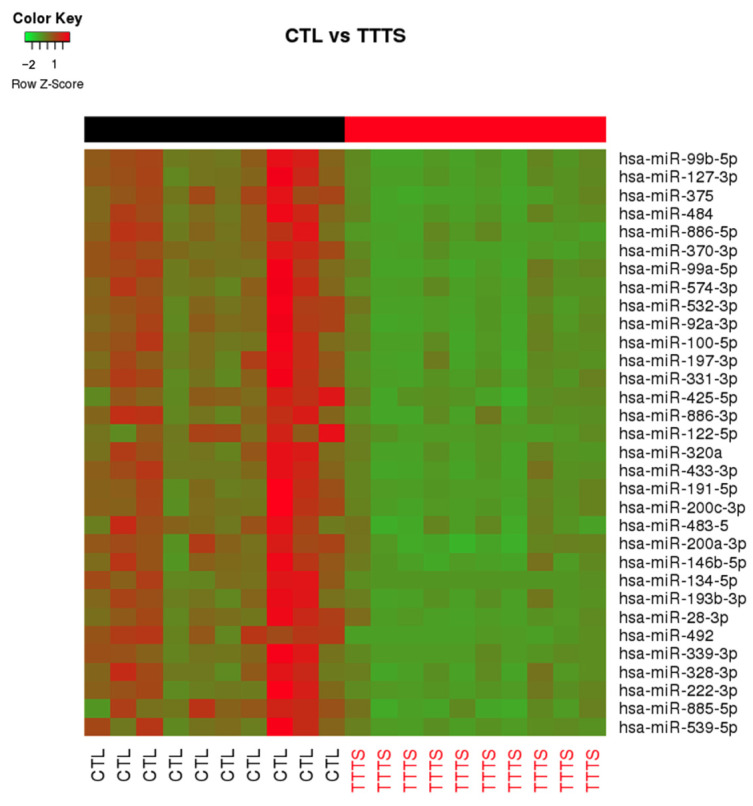
Heat map based on *t*-test from arrays separated TTTS subjects (red labels, *n* = 10) from controls (black labels, *n* = 10). Green indicates down-regulation; red, up-regulation. miRNAs in the right-hand column are ranked by *t*-test q-value.

**Figure 2:**
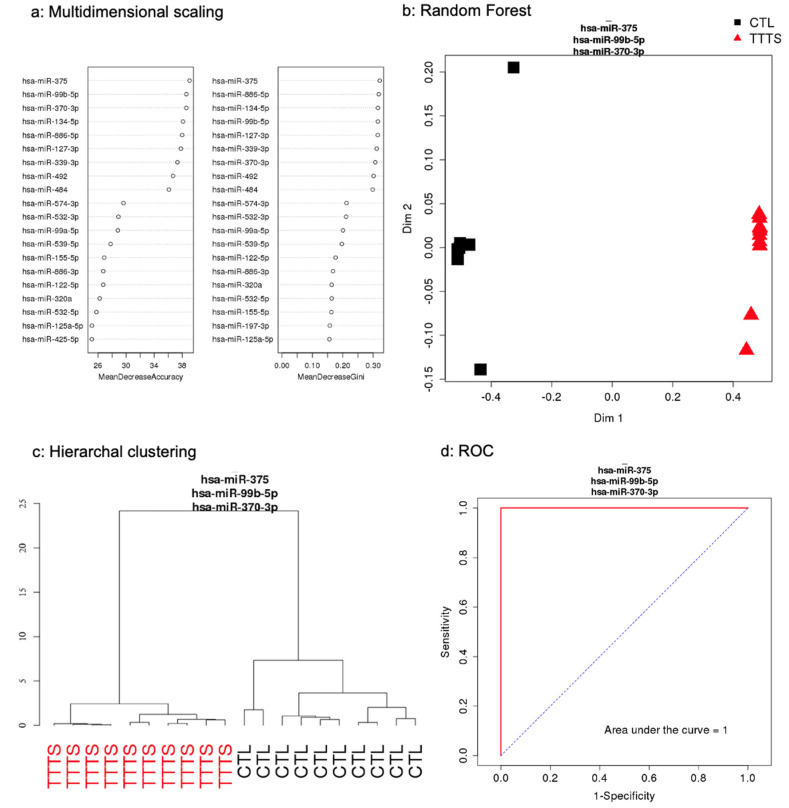
Amniotic fluid miRNAs differentiated TTTS subjects (*n* = 10) from singleton controls (*n* = 10). (**a**) Rank of most important miRNAs by multidimensional scaling. (**b**) Random forest (RF) analysis demonstrated that miR-99b, miR-370 and miR-375-3p differentiated the two groups. (**c**) Hierarchal clustering showed separation between the TTTS and controls. (**d**) Receiver operating curves using the top 3 miRNAs showed an area under the curve of 1.

**Figure 3:**
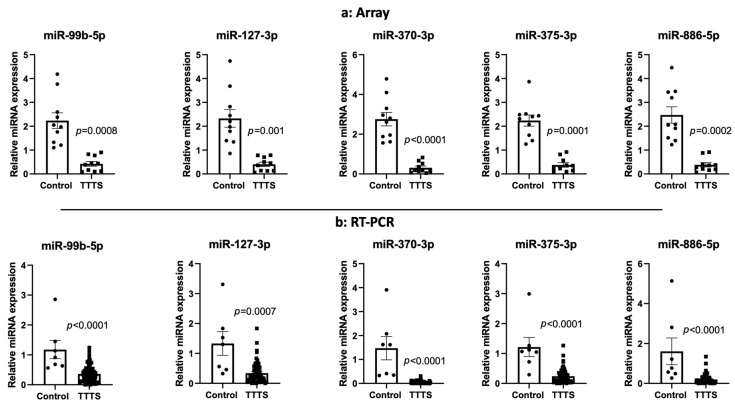
Bar graphs of 5 differentially expressed miRNAs from miRNA arrays (**a**) and RT-PCR (**b**). *p*-values displayed reflect individual comparisons. Individual miRNAs selected based on *q* < 0.0025. (**a**) Relative miRNA expression of select miRNAs among TTTS subjects *n* = 10 and singleton controls *n* = 10. (**b**) Relative miRNA expression of select miRNAs by RT-PCR utilizing miRNA-363 as a normalizer. TTTS *n* = 50, singleton controls *n* = 7.

As shown in Figure 1, using a stringent *q* < 0.0025, we detected 32 miRNAs differentially expressed between the two groups (Table 2). Expression of all miRNAs was downregulated in the TTTS group when compared to the control group. The rank of most important miRNAs as determined by the RF multidimension scaling of the estimated proximity matrix plots is displayed in Figure 2A. Our top three differentiating miRNAs were miR-99b, miR-370 and miR-375. RF analysis using these miRNAs (Figure 2B) demonstrated stark differentiation between the TTTS subjects and the singleton controls. Hierarchal clustering effectively separated the TTTS subjects and singleton controls using these miRNAs (Figure 2C). A receiver operating curve (ROC) was generated based on miR-99b, miR-370 and miR-375 and shows 100% sensitivity and specificity to distinguish TTTS from control (Figure 2D). Box plots were generated for miRNA-99b-5p, miR-127-3p, miR-370-3p, miR-375-3p and miR-886-5p (Figure 3A) and show statistically significant differences with *p* < 0.002. 

### 3.3. Degradation Results

Subject 1 had Stage IIIC TTTS with RV Tei index of 0.58 and LV Tei index of 0.54 at 18 weeks and 2 days gestation. Subject 2 was a monochorionic-diamniotic twin pair at 18 weeks and 4 days with Stage IV TTTS. The RV Tei index was 0.99 and the LV Tei index was 0.79.

Arrays were performed on six amniotic fluid aliquots as described. Correlation plot analysis (Supplemental Figure S1a,b) showed the threshold cycle (Ct) levels from the aliquots exposed to room temperature were not different from those immediately placed on ice (correlation *p* < 0.0001). Hierarchal clustering showed the 4-h and 24-h aliquots were more similar to each other than the immediate ice aliquot in subject 1 (Supplemental Figure S1c). In subject 2, immediate ice and 24-h aliquots were more similar to each other than the 4-h aliquot (Supplemental Figure S1d).

### 3.4. RT-PCR Results

To confirm array findings, RT-PCR was performed on 57 subjects (10 TTTS discovery cohort subjects, 7 controls subjects, 40 distinct TTTS subjects of the validation cohort, as denoted in Table 1) targeting six miRNAs. miRNAs selected for RT-PCR confirmation were miR-370-3p, miR-375-3p, miR-886-5p, miR-127-3p and miR-99b-5p. miR-363 was used as a normalizer. Differentially expressed miRNAs by array data also showed consistent differential expression when assessed by RT-PCR (Figure 3b). 

### 3.5. Clinical Association with Specific miRNAs

To determine if specific miRNAs are associated with clinical findings or fetal survival, we subdivided our TTTS population based on clinical characteristics and compared expression of miR-370-3p, miR-375-3p, miR-886-5p, miR-127-3p and miR-99b-5p by RT-PCR between groups. Comparison groups were TTTS subjects (1) with demise of either or both fetuses before delivery versus those who both survived, (2) presence versus absence of fetal growth restriction prior to SFLP, (3) with moderate or severe tricuspid regurgitation (TR) versus those with mild or no TR prior to SFLP and (4) with moderate or severe ventricular hypertrophy versus those with mild or no hypertrophy prior to SFLP. 

**Figure 4:**
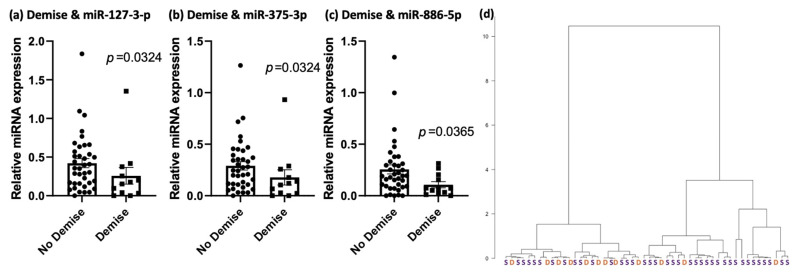
(**a**–**c**) Bar graphs comparing relative expression of miRNA-127-3p (*p* = 0.0324), miRNA-375-3p (*p* = 0.0324) and miRNA-886-5p (*p* = 0.0365) between TTTS fetuses with demise versus survival to delivery (no demise). (**d**) Hierarchal cluster demonstrating the sorting of TTTS subjects by demise status (demise shown as orange ‘D’, survival as purple ‘S’).

We found fetuses with demise (*n* = 12) had lower expression of miRNA-127-3p (*p* = 0.0324), miRNA-375-3p (*p* = 0.0324) and miRNA-886-5p (*p* = 0.0365) compared to those who survived to delivery (*n* = 38) (Figure 4a–c). Using these three miRNAs, hierarchal clustering revealed some separation between the two groups (Figure 4d). The majority of demises clustered to the left-hand side of the Figure 4d. The three demised subjects that clustered to the right-hand side of Figure 4d were subject numbers 36, 50 and 34, respectively (see Supplementary material for clinical details of these cases). Based on the number of demises, receiver operator curves were not sufficiently powered to determine sensitivity and specificity of this comparison. 

There were no significant differences in these miRNAs between TTTS fetuses with more severe tricuspid regurgitation or ventricular hypertrophy at the time of the pre-SFLP echocardiogram, however these findings can be subjective. Fetal gestational age, sex or the presence or absence of fetal growth restriction also did not show differential expression (not shown). 

### 3.6. Network Analysis

**Figure 5:**
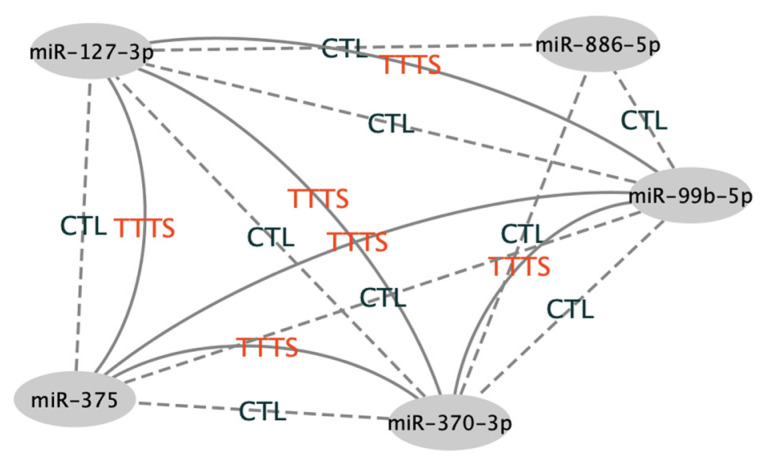
Network analysis.

Network analysis was used to determine the relationship of miR-99b-5p, miR-127-3p, miR-375, miR-886-5p and miR-370-3p. As shown in Figure 5, a correlation was observed between all miRNAs in TTTS subjects with the exception of miR-886-5p. In control subjects, miR-886-5p correlated with miR-370, miR-127-3 and miR-99b. Neither controls nor TTTS subjects had a correlation between miR-886-5 and miR-375.

### 3.7. Pathway Analysis

Pathway analysis of our 32 differentially expressed miRNAs demonstrated over-representation in pathways involving angiogenesis (VEGF signaling, fluid shear stress), inflammation (Toll-like receptor pathway) and activation of the proteosome (Table 3 and expanded depicting miRs for each of the putative pathways in Supplemental Table S2). Pathway analysis of the miRNAs were investigated with RT-PCR showed over-representation in KEGG pathways involving folate biosynthesis, fatty acid degradation and galactose metabolism.

## 4. Discussion

This is the first comprehensive comparison of amniotic fluid miRNAs in TTTS to normal controls. This design is an intentional exploration to determine if amniotic fluid miRNAs have potential as a biomarker for TTTS. We identified several miRNAs that are highly differentially expressed between TTTS recipients and controls. While the presence of differences given our use of singleton controls was anticipated, the particular miRNAs with differential expression may nonetheless yield insight into the pathophysiology of TTTS, recipient cardiomyopathy and fetal demise. We identified miRNA-127-3p, miRNA-375-3p and miRNA-886-5p as potential biomarkers of future fetal demise. Additionally, we successfully demonstrated fidelity between array and RT-PCR expression of miRNAs and provide proof of viable methodology using amniotic fluid collected at clinically relevant timepoints for assessment of miRNAs. Finally, we demonstrate that amniotic fluid miRNAs are stable at room temperature for several hours, enhancing the opportunity to utilize a wide range of clinically collected samples. 

### 4.1. Principal Results

Several miRNAs showed highly significantly differences when comparing TTTS and singletons, with the top 3 differentially expressed miRNAs being miR-99b-5p, miR-375-3p and miR-370-3p. 

Previous studies have shown miRNA-99b-5p plays an important role in endothelial cell differentiation and may regulate angiogenesis [23]. Maternal circulating levels of miR-99b-5p have been associated with gestational age-adjusted birth weight z-score and it has been found to be downregulated in the placenta of growth-restricted monochorionic twins [24,25]. Together, these findings point to an important role of miRNA-99b-5p in placental development. Interestingly, miR-375-3p seems important in congenital heart disease (CHD) as it has a role in cardiac dysfunction and in cardiogenesis. miR-375 upregulation appears protective against hypoxia-induced apoptosis of cardiomyocytes via forkhead box P1 (FOXP1) and Bcl like protein 2 (Bcl2l2) upregulation, which may be associated with the observed lower expression of miR-375 in TTTS who had later demise (*p* = 0.0324). Differences in miR-375-3p expression have been demonstrated in maternal serum of those affected by fetal CHD, suggesting it may be a potential biomarker [26]. KEGG analysis of miR-99p and miR-375-3p suggest they may be involved in folate biosynthesis and aberrations in placental development [27,28,29]. miR-370 can regulate angiogenic activity of endothelial cells by targeting smoothened (SMO) and bone morphogenetic protein-2 (BMP-2) [30]. In addition, miR-370 may play an important role in the cardiac myocyte response to oxidative stress [31,32]. miR-375-3p was upregulated in hearts of rats subjected to transverse aortic constriction and in angiotensin II (Ang II)-induced primary cardiomyocyte hypertrophy model. It therefore appears important in angiotensin-mediated promotion of cardiac hypertrophy [33]. 

Beyond our top 3 differentiating miRNAs, many of our additional differentially expressed miRNAs (Table 2) also have known involvement in cardiovascular disease, angiogenesis and placental pathologies, which may play an important role in TTTS. 

Array and RT-PCR confirmation identified miR-127-3p to be downregulated in TTTS. We found that it is significantly more downregulated in TTTS subjects who go on to experience later demise (*p* = 0.0324). miR-127 appears to regulate expression of retrotransposon-like gene RTL1, which has a key role in placenta formation [34,35,36] and has been shown to have abnormal methylation in fetal growth restriction [37].

We also found miR-886-5p to be differentially expressed and associated with later demise. Prior work has found miR-886-5p to be upregulated in early senescence of endothelial cells [38], and lower miR-886 expression was associated with preterm birth [39]. 

Within the realm of cardiovascular disease, we found differential expression of miR-484, miR-433, miR-320 and miR-383-5p, all of which have previously been documented to play an important role in the heart’s response to ischemia/reperfusion injury [40,41,42,43]. Additionally, miR-484-5p and miR-484-3p are suggested as potential therapeutic targets to limit hypoxia-induced myocardial injury [44,45]. Other differentially expressed miRNAs are also associated with cardiac disease; miR-134 is elevated in early stages of myocardial infarction and its inhibition can decrease apoptosis of cardiomyocytes [46,47]. miR-492 is also suggested as a biomarker in adult acute myocardial infarction [48]. Downregulation of miR-200a can protect cardiomyocytes against injury and apoptosis after infarction or doxorubicin-stress [49,50]. Differences in miR-146b expression have been shown in the cardiac muscle of infants with right ventricular outflow tract obstruction undergoing surgery and its inhibition may increase hypoxia-induced apoptosis [51]. Others found miR-146b targets Notch1 and protected cardiomyocytes against inflammation and apoptosis [52]. miR-433 is a potential target to prevent/decrease progression of cardiac fibrosis via TGFβ and Smad-3 pathway [53]. miR-222 regulates protective cardiac response to exercise (in rats) and is also involved in a broad variety of physiologic and pathologic functions [54,55]. Additionally, miR-100, miR-92a, miR-320a, miR-122 have also previously been shown to be impacted by adult cardiomyopathy [56,57]. 

Our additional differentially expressed miRNAs included several that are relevant to angiogenesis and placental function. miR-492 exerts an anti-angiogenic activity on endothelial cells and has gained attention as a potential therapeutic anti-angiogenic agent [58]. miR-191 suppresses angiogenesis by activation of NFkB-signaling [59]. miR-200c and miR-222 appear important in placental trophoblast proliferation, migration and apoptosis and are abnormally expressed in pre-eclampsia [60,61]. miR-885-5p is upregulated in pre-eclampsia compared to healthy controls [62]. 

Our network analysis demonstrated a unique relationship of miR-886-5p to miR-127, -370-3p and -99b only among controls. In a human umbilical vein endothelial cell model, miR-886-5p was important in signaling early senescence [38]. This suggests that senescent signals may be further downregulated in TTTS, in favor of pro-angiogenic forces.

### 4.2. Clinical Implications

The clinical management of monochorionic pregnancies involves labor-intensive monitoring for the development of TTTS. In addition, the staging, prognostication and timing of intervention of TTTS remains clinically challenging. A TTTS biomarker could therefore be a powerful tool to assist in clinical management. A previous small study by Mackie et al. demonstrated no miRNA differences when examining maternal circulating miRNAs in TTTS pregnancies compared to uncomplicated monochorionic pregnancies [63].

Early TTTS is sometimes treated initially with amnioreduction [2], and amniotic fluid miRNA testing could be employed diagnostically or prognostically at that time. More study is needed to define the miRNA profile of uncomplicated monochorionic pregnancies and identify miRNAs that portend progression to severe TTTS.

This is the first study demonstrating the stability of miRNAs in amniotic fluid collected at the time of clinically indicated procedures, fidelity between miRNA expression by array and RT-PCR, and significant amniotic fluid miRNA differences in the TTTS population compared to control. 

Perhaps most notably, we identify three miRNAs that are associated with fetal demise. This finding requires the attention of future investigations, as our study was underpowered for this analysis, with only 12 demises. Additionally, we included all demises regardless of possible etiology of demise. 

### 4.3. Research Implications

As we gain a better understanding of specific miRNAs and their targets, amniotic fluid miRNAs may provide valuable insight into monochorionicity, placental pathology, IUGR, hypertensive cardiomyopathy and TTTS. Further studies on miRNA characterization may provide valuable insight into early TTTS development.

### 4.4. Strengths and Limitations

The strengths of our study include the large sample size of TTTS patients and the RT-PCR confirmation of our findings. Our findings are robust, with many highly significant miRNA differences. We find that the exploration of amniotic fluid miRNAs using wide-spectrum, array technology is a valid methodology. 

The most significant limitation of this study is use of singleton fetuses for our control population. Unfortunately, we did not have amniotic fluid samples from non-TTTS twin pregnancies available for this study. In the current era of noninvasive genetic pregnancy screening (NIPS), amniocentesis is rarely performed. While imperfect, we believe this comparison was a necessary first step to discover the potential of amniotic fluid miRNAs as a viable biomarker.

Variability of amniotic fluid handling conditions between our TTTS and control populations is an additional limitation of our study. Our degradation experiment was carefully designed to assess miRNA stability in AF over similar handling conditions and demonstrates our results to be sound. Furthermore, we observed a decrease in levels of AF miRNAs in TTTS, again supporting the findings that control AF miRNAs were not degraded, regardless of temporary storage at room temperature. 

The source of the miRNAs of interest is unknown. All TTTS samples were obtained from the recipient twin amniotic fluid space. Although shared circulation between twins defines TTTS, it remains unknown if the recipient and donor twin amniotic fluid miRNA profiles would differ. Sampling the donor space would involve a higher risk of fetal injury due to the presence of oligohydramnios and is therefore not performed. 

Additionally, we recognize we cannot determine if these miRNAs contribute to the cause of disease or if they are simply biomarkers of biological changes altered in response to TTTS. In an attempt to understand this question, we performed logistic regression of these miRs. Although a subset was significant by logistic regression (*p*-value), none were significant by q-value (not shown). Since there are no animal models of TTTS, we feel this analysis is too preliminary to suggest these miRs may be causative of the disease. 

Lastly, miR-886-5p was identified as a vault RNA in 2009, its pre-miRNA was suggested to function as a non-coding RNA [64], and it has been identified as a biomarker miR [65,66] and a functional miR in more recent papers [67,68]. Due to its recent role as a functional miR, we included it in the manuscript. 

## 5. Conclusions

Our results demonstrated that amniotic fluid miRNAs have potential as a novel biomarker for the diagnosis and prognostication of TTTS. We identified many miRNAs that are highly differentially expressed between TTTS recipients with cardiomyopathy and singleton controls; with the top-3 miRNAs being miR-99b, miR-370-3p and miR-375-3p. Our results are reproducible between array and RT-PCR methodologies, even when including a distinct validation cohort of patients. We found miR-127-3p, miR-375-3p and miR-886-5p to be additionally downregulated in TTTS cases that would have future demise. Many of the differentially-expressed miRNAs in TTTS are involved in angiogenesis and cardiac adaption to stress. While more study is needed, this opens a new opportunity to increase our understanding of this serious disease and the developing heart’s response to stress. With further study, amniotic fluid miRNAs could potentially fulfill a powerful diagnostic and prognostic role in the clinical management of TTTS and elucidate a complex pathophysiologic process. 

## Figures and Tables

**Table 1:** Subject characteristics.

Subject No.	Diagnosis	Sex	GA (Weeks)	Donor Growth Restriction	Donor Demise	Recipient RV Tei Index	Recipient LV Tei Index
**TTTS Discovery Cohort**							
1	TTTS	M	23.71	Y, severe	N	1.36	0.70
2	TTTS	M	18.71	Y, severe	N	1.28	0.85
3	TTTS	M	20.71	Y, severe	Y	0.91	0.54
4	TTTS	F	22.71	Y, severe	N	0.87	0.63
5	TTTS	F	23.71	Y, severe	N	0.83	0.57
6	TTTS	M	17.29	N	Y	0.77	0.62
7	TTTS	M	17.43	N	Y	0.75	0.67
8	TTTS	F	21.43	Y, severe	N	0.70	0.43
9	TTTS	F	18.29	N	N	0.69	0.67
10	TTTS	M	17.43	N	N	0.68	0.66
**Singleton Control Subjects**							
11 ^†^	AMA	F	18.29	NA	NA	NA	NA
12	Incompetent cervix	M	22.86	NA	NA	NA	NA
13 ^†^	Incompetent cervix	M	23.00	NA	NA	NA	NA
14	Unreportable NIPT	M	18.86	NA	NA	NA	NA
15 ^†^	AMA	F	16.86	NA	NA	NA	NA
16	Borderline NIPT	F	16.57	NA	NA	NA	NA
17 ^†^	AMA	M	16.29	NA	NA	NA	NA
18 ^†^	positive NIPT	M	20.57	NA	NA	NA	NA
19 ^†^	AMA, Thalassemia carrier	F	22.71	NA	NA	NA	NA
20 ^†^	inconclusive NIPT	M	20.00	NA	NA	NA	NA
**TTTS validation cohort**							
21	TTTS	UK	18.57	Y, severe	Y	0.59	0.64
22	TTTS	UK	17.29	N	N	0.69	0.58
23	TTTS	F	20.57	Y, severe	Y	0.61	0.74
24	TTTS	M	22.71	N	N	0.65	0.53
25	TTTS	F	17.43	Y	N	0.56	0.53
26	TTTS	M	17.29	N	N	0.62	0.47
27	TTTS	M	18.71	N	N	0.57	0.66
28	TTTS	M	18.57	N	N	1.10	0.89
29	TTTS	M	21.00	Y	N	0.78	0.68
30	TTTS	M	19.43	Y, severe	N	0.58	0.52
31	TTTS	F	20.43	Y, severe	N	0.72	0.81
32	TTTS	F	23.14	N	N	0.66	0.73
33	TTTS	F	16.43	N	N	0.73	0.69
34	TTTS	M	21.00	Y, severe	Y	0.65	0.68
35	TTTS	M	18.57	Y, severe	N	0.66	0.79
36	TTTS	M	21.29	Y, severe	Y	0.63	0.64
37	TTTS	F	25.43	N	N	0.78	0.53
38	TTTS	M	17.14	Y	N	0.75	0.47
39	TTTS	F	21.14	Y, severe	N	0.64	0.62
40	TTTS	F	18.71	Y, severe	N	0.68	0.47
41	TTTS	M	24.86	Y, severe	N	0.66	0.58
42	TTTS	F	16.86	Y, severe	N	0.55	0.56
43	TTTS	F	20.71	Y	N	0.59	0.60
44	TTTS	M	18.14	Y	Y	0.51	0.65
45	TTTS	F	18.29	N	N	0.55	0.60
46	TTTS	M	20.71	Y, severe	N	0.64	0.54
47	TTTS	M	16.71	Y, severe	N	0.57	0.62
48	TTTS	F	22.29	Y, severe	N	0.65	0.64
49	TTTS	M	25.14	Y, severe	Y	0.67	0.56
50	TTTS	F	20.00	Y, severe	Y	0.63	0.68
51	TTTS	F	17.57	Y, severe	N	0.63	0.49
52	TTTS	F	25.86	N	N	1.31	0.64
53	TTTS	F	17.57	Y	N	0.69	0.40
54	TTTS	M	19.14	Y, severe	N	0.67	0.55
55	TTTS	M	16.86	N	N	0.56	0.51
56	TTTS	M	20.43	N	N	0.61	0.58
57	TTTS	M	20.14	Y, severe	N	0.64	0.60
58	TTTS	M	21.43	Y, severe	N	0.82	0.69
59	TTTS, double demise	F	18.86	Y, severe	Y	1.90	1.50
60	TTTS, isolated recipient demise	F	23.14	Y, severe	N	0.57	0.53

^†^ Denotes subject was included in the RT-PCR portion of experiment. Abbreviations: GA: gestational age; RV: right ventricle; LV: left ventricle; in reference to fetal sex, M: male; F: female, UK: unknown; TTTS: twin-twin transfusion syndrome; AMA: advanced maternal age. NIPT: non-invasive prenatal testing.

**Table 2:** Differentially expressed miRNAs by array.

miRNA	Fold Change	Q Value (by Wilcox *t*-test)	Control Sample Mean	Control Sample Standard Deviation	TTTS Sample Mean	TTTS Standard Deviation
hsa-miR-99b-5p	−1.8113	0.00059	2.2353	1.0434	0.4240	0.3162
hsa-miR-127-3p	−1.9207	0.00059	2.3263	1.1693	0.4056	0.2850
hsa-miR-375-3p	−1.8611	0.00059	2.2378	0.7424	0.3767	0.2982
hsa-miR-484	−1.7135	0.00059	2.1603	1.0494	0.4468	0.3206
hsa-miR-886-5p	−2.0864	0.00059	2.4716	1.0864	0.3852	0.2902
hsa-miR-370-3p	−2.4452	0.00059	2.7575	1.0742	0.3123	0.2730
hsa-miR-99a-5p	−1.8466	0.00078	2.3329	1.1180	0.4863	0.4627
hsa-miR-574-3p	−1.5480	0.00078	1.9632	1.0388	0.4151	0.2695
hsa-miR-532-3p	−1.8939	0.00078	2.3093	1.1001	0.4154	0.3966
hsa-miR-92a-3p	−1.5980	0.00083	2.0174	0.8811	0.4194	0.3155
hsa-miR-100-5p	−1.5639	0.00083	2.0255	1.0740	0.4616	0.3513
hsa-miR-197-3p	−1.4270	0.00083	1.9019	0.8623	0.4749	0.3085
hsa-miR-331-3p	−1.6085	0.00083	2.0384	1.0950	0.4299	0.2792
hsa-miR-425-5p	−1.1909	0.00083	1.7537	0.7842	0.5628	0.2567
hsa-miR-886-3p	−1.6453	0.00083	2.1731	1.0641	0.5278	0.3521
hsa-miR-122-5p	−2.3081	0.00083	2.6098	1.5017	0.3017	0.3264
hsa-miR-320a-3p	−1.6845	0.00083	2.1511	1.1301	0.4666	0.3506
hsa-miR-433-3p	−1.8726	0.00117	2.3578	1.1409	0.4852	0.4784
hsa-miR-191-5p	−1.5862	0.00117	1.9796	1.2070	0.3934	0.2882
hsa-miR-200c-3p	−1.4516	0.00117	1.8773	1.0379	0.4257	0.3064
hsa-miR-483-5p	−1.1983	0.00117	1.7824	0.7386	0.5842	0.3740
hsa-miR-200a-3p	−0.9514	0.00162	1.5064	0.4623	0.5550	0.2976
hsa-miR-146b3p	−1.3653	0.00162	1.7593	0.8855	0.3940	0.3724
hsa-miR-134-5p	−1.3302	0.00162	1.3617	0.8735	0.0315	0.0673
hsa-miR-193b-5p	−1.6562	0.00162	2.1416	1.2328	0.4854	0.3894
hsa-miR-28-3p	−1.6422	0.00162	2.0473	1.0659	0.4051	0.3930
hsa-miR-492	−1.5150	0.00180	1.5784	0.6285	0.0634	0.1017
hsa-miR-339-3p	−1.3255	0.00189	1.4845	0.9884	0.1590	0.1812
hsa-miR-328-3p	−1.4769	0.00216	2.0236	1.0648	0.5467	0.4001
hsa-miR-222-3p	−1.6972	0.00216	2.0848	1.4409	0.3876	0.3224
hsa-miR-885-5p	−1.3903	0.00216	1.8579	0.7969	0.4676	0.3943
hsa-miR-539-5p	−1.5847	0.00249	1.8534	1.2129	0.2686	0.2365

**Table 3:** Putative pathways affected by significantly altered miRNAs.

*p*-Value	Q-Value	Category
0.000114	0.0052579	VEGF signaling pathway, possibly involved in angiogenesis
0.000179	0.0071438	Fluid shear stress, atherosclerosis, possibly related to vascular dysfunction.
0.000265	0.0085739	Fatty acid biosynthesis
0.000398	0.011679	Phospholipase D signaling pathway, likely involved in cell division
0.000503	0.0125707	Longevity regulated pathway in multiple species, likely involving the proteasome through PI2k/AKT/TOR
0.000521	0.0125707	Peroxisome
0.000615	0.0125707	Proteosome
0.000684	0.0125707	Thyroid hormone signaling pathway
0.000704	0.0125707	Neurotrophin signaling pathway
0.000719	0.0125707	Toll-like receptor signaling pathway, possibly involved in inflammation
0.000967	0.0125953	Aminoacyl-tRNA biosynthesis

## Data Availability

The data that support the findings of this study are available from the corresponding author upon reasonable request.

## Supplementary Materials

The following supporting information can be downloaded at https://www.mdpi.com/article/10.3390/jcdd9020037/s1, Supplemental text reviewing the methods and results of the degradation experiment, Figure S1: Degradation experiment results, Table S1: Fetal demise information, Table S2: Pathway analysis.
