# Supplementary material for: Amniotic Fluid microRNA in Severe Twin-Twin Transfusion Syndrome Cardiomyopathy—Identification of Differences and Predicting Demise
Source: J Cardiovasc Dev Dis. 2022 Jan 23;9(2):37. doi: 10.3390/jcdd9020037 (PMC8878714; doi:10.3390/jcdd9020037)
Supplement: Supplementary file 1 [file jcdd-09-00037-s001.zip › jcdd-1536124-supplementary.pdf]

Supplementary Materials

1. Degradation Experiment

1.1. Background

Control amniotic fluid was obtained for clinical karyotyping and was therefore exposed to storage at room temperature for varying durations (4 to 22 hours). This experiment was designed to assess the stability of miRNAs in AF exposed to similar conditions.

1.2. Methods

Prospectively, two patients with Twin-twin transfusion syndrome (TTTS) undergoing selective fetoscopic laser photocoagulation (SFLP) who had consented to participate in the study were selected. Amniotic fluid from these patients was sampled per usual procedure. Fluid was aliquoted and exposed to 3 conditions: (1) immediate placement on ice, spun and frozen to -80°C per usual procedure for TTTS samples, (2) maintained at room temperature for 4 hours before being spun and frozen to -20°C, (3) maintained at room temperature for 24 hours before being spun and frozen to -20°C. From these 6 samples, RNA extraction, miRNA arrays and data analysis were performed per procedure described elsewhere in Methods.

1.3. Results

Subject 1 of this experiment was a triplet pregnancy with a monochorionic-diamniotic twin pair and a dichorionic bystander triplet at 18 weeks and 2 days gestation. The monochorionic-diamniotic twin pair had Stage IIIC TTTS with RV Tei index of 0.58 and LV Tei index of 0.54. This subject experienced a donor fetus demise post-procedure. Subject 2 of this experiment had monochorionic-diamniotic twins at 18 weeks and 4 days and had Stage IV TTTS. The RV Tei index was 0.99 and the LV Tei index was 0.79. Both twins survived, though the recipient later developed pulmonary valve stenosis.

To determine if amniotic fluid miRNAs are stable at room temperature, arrays were performed on six amniotic fluid aliquots as described. Correlation plot analysis (Supplemental Figure 1a and 1b) from arrays showed the threshold cycle (Ct) levels from the aliquots exposed to room temperature were not different from those immediately placed on ice (correlation $p<0.0001$), regardless of patient sample. Hierarchal clustering showed the 4-hour and 24-hour aliquots were more similar to each other than the immediate ice aliquot in subject 1 (S1c). In subject 2, immediate ice and 24-hour aliquots were more similar to each other than the 4 hour aliquot (S1d).

1.4. Conclusions

Amniotic fluid miRNAs are stable at room temperature for up to 24 hours.

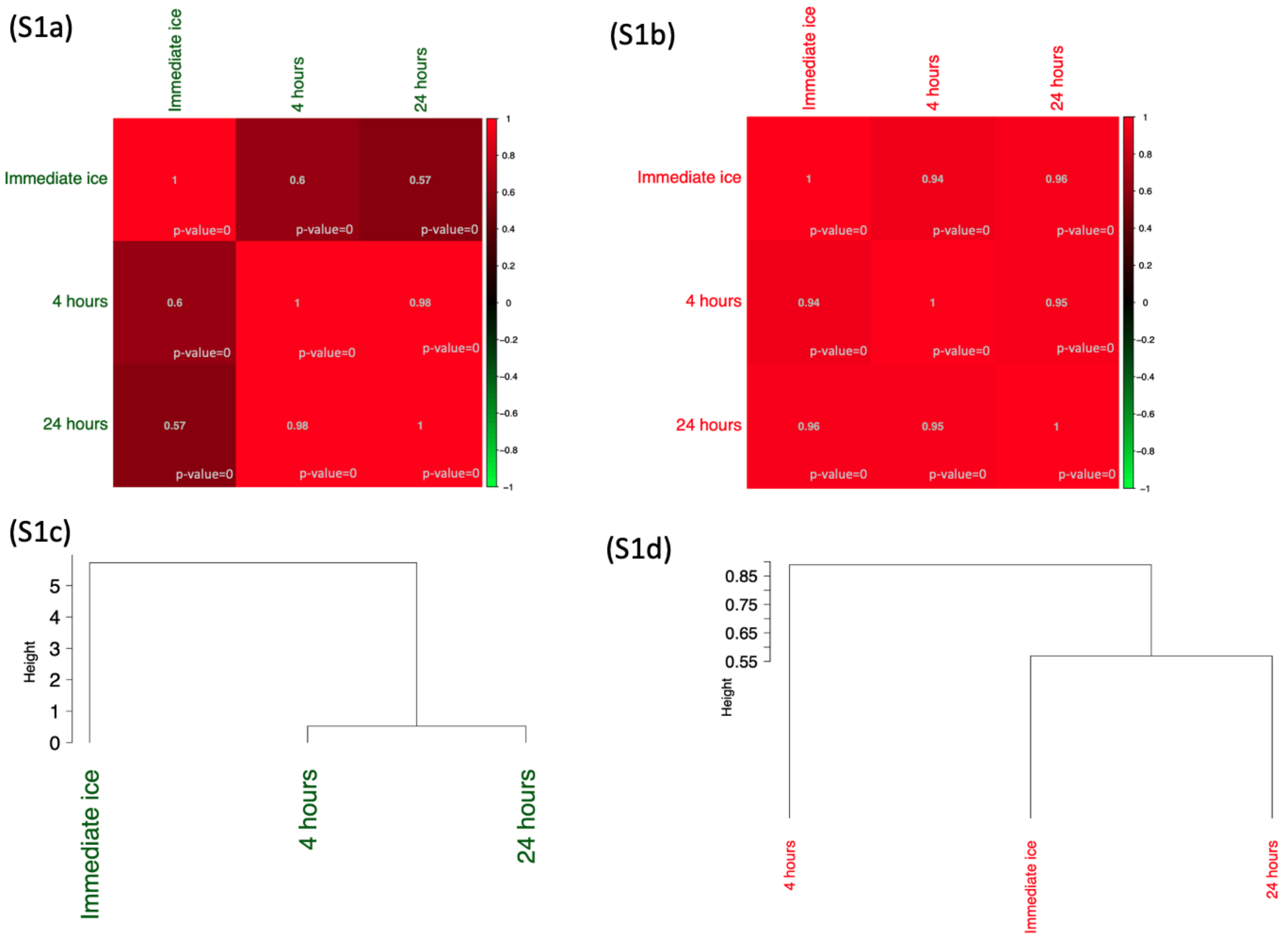

Supplemental Figure S1: Degradation experiment results. (a) and (b): Correlation plot analysis showed similar Ct levels regardless of how the aliquot handled (p<0.0001). (c): Hierarchal clustering for subject 1 showed a greater similarity between the 4-hour and 24-hour aliquots than the immediate ice aliquot. (d): In subject 2, the immediate ice and 24-hour aliquots were more similar to each other than to the 4-hour aliquot. Parts (a) and (c) show results for subject 1. Parts (b) and (d) show results subject 2.

2. Fetal Demise Information

Supplemental Table S1: Available Clinical Information Regarding Subjects with Fetal Demise

Subject no.	Clinical Information
3	After SFLP, fetuses developed TAPS. Donor became hydropic and then had demise. Records also indicated a cord accident was suspected. Placental share was 70/30. Viable recipient twin delivered at 30 weeks.
6	Placental share was 70/30. Timing of donor demise was unknown.
7	Placental share was 60/40. Donor demised POD #3.
21	Donor experienced demise at 22 weeks (almost 3.5 weeks after SFLP)
23	Placental share was 90/10.

34*	Donor demise POD #1 after SFLP, thought to be due to acute placental insufficiency. Donor placenta share was 10% and had a membranous cord insertion. Donor had low heart rate at end of SFLP procedure. Laser time was long at 8.5 minutes.
36*	Placental share was 70/30. Laser time was longer at 5 minutes. Timing of demise was unknown.
44	Triplets with a mo-di set and a dichorionic bystander. Placental share was 90/10.
49	Placental share was 80/20. Timing of demise unknown. Laser time 5.5 minutes.
50*	Intertwin membrane was disrupted, twin set became monoamniotic. Demise occurred POD #28, possibly due to cord accident. Due to monoamniotic status, unknown if demised twin was recipient or donor.
59	This twin set had very severe cardiomyopathy with the highest Tei values seen in the entire cohort. Demise occurred on POD #1.
60	Isolated recipient demise occurred on POD #1 from SFLP.

Note that all fetuses experiencing a demise were included, regardless of possible demise mechanism.
* Indicates subject segregated to the right-hand side of hierarchal cluster seen in Figure 4d, apart for majority of demises.
Abbreviations: SFLP: selective fetoscopic laser photocoagulation, TAPS: twin anemia polycythemia sequence, POD: post-operative day.

3. Pathway analysis: Several differentially expressed miRNAs (from Table 2) were over-represented in the following pathways, which may be of interest in TTTS. A p-value of < 0.001 was used to determine significance.

Supplemental Table S2:

miRNAs	p-value	Q-value	Category
miR-99b-5p, miR-375-3p, miR-484, miR-370-3p; miR-99a-5p; miR-574-3p; miR-532-3p; miR-92a-3p; miR-100-5p; miR-197-3p; miR-331-3p; miR-425-5p; miR-122-5p; miR-320a-3p; miR-433-3p; miR-200c-3p; miR-483-5p; miR-200a-3p; miR-134-5p; miR-193b-5p; miR-492; miR-222-3p; miR-885-5p	0.000114	0.0052579	VEGF signaling pathway, possibly involved in angiogenesis
miR-99b-5p; miR-375-3p; miR-484; miR-370-3p; miR-99a-5p; miR-574-3p; miR-532-3p; miR-92a-3p; miR-100-5p; miR-197-3p; miR-331-3p; miR-425-5p; miR-122-5p; miR-320a-3p; miR-433-3p; miR-191-5p; miR-200c-3p; miR-483-5p; miR-200a-3p; miR-146b-3p; miR-134-5p; miR-193b-5p; miR-28-3p; miR-339-3p; miR-328-3p; miR-222-3p; miR-885-5p; miR-539-5p	0.000179	0.0071438	Fluid shear stress, atherosclerosis, possibly related to vascular dysfunction.
miR-375-3p; miR-484; miR-532-3p; miR-92a-3p; miR-100-5p; miR-197-3p; miR-331-3p; miR-425-5p; miR-191-5p; miR-200c-3p; miR-222-3p; miR-539-5p	0.000265	0.0085739	Fatty acid biosynthesis
miR-99b-5p; miR-127-3p; miR-375-3p; miR-484; miR-370-3p; miR-99a-5p; miR-574-3p; miR-532-3p; miR-92a-3p; miR-100-5p; miR-197-3p; miR-331-3p; miR-425-5p; miR-122-5p; miR-320a-3p; miR-433-3p; miR-191-5p; miR-200c-3p; miR-483-5p; miR-200a-3p; miR-146b-3p; miR-134-5p; miR-193b-5p; miR-492; miR-222-3p; miR-885-5p	0.000398	0.011679	Phospholipase D signaling pathway, likely involved in cell division

miR-99b-5p; miR-375-3p; miR-484; miR-370-3p; miR-99a-5p; miR-532-3p; miR-92a-3p; miR-100-5p; miR-197-3p; miR-331-3p; miR-425-5p; miR-122-5p; miR-320a-3p; miR-433-3p; miR-191-5p; miR-200c-3p; miR-200a-3p; miR-134-5p; miR-492; miR-339-3p; miR-328-3p; miR-222-3p; miR-885-5p; miR-539-5p	0.000503	0.0125707	Longevity regulated pathway in multiple species, likely involving the proteasome through PI2k/AKT/TOR
miR-375-3p; miR-484; miR-370-3p; miR-532-3p; miR-92a-3p; miR-100-5p; miR-197-3p; miR-331-3p; miR-425-5p; miR-122-5p; miR-320a-3p; miR-191-5p; miR-200c-3p; miR-483-5p; miR-200a-3p; miR-134-5p; miR-193b-5p; miR-492; miR-328-3p; miR-222-3p; miR-885-5p; miR-539-5p	0.000521	0.0125707	Peroxisome
miR-375-3p; miR-484; miR-99a-5p; miR-532-3p; miR-92a-3p; miR-100-5p; miR-331-3p; miR-425-5p; miR-122-5p; miR-320a-3p; miR-191-5p; miR-200a-3p; miR-193b-5p; miR-222-3p; miR-539-5p	0.000615	0.0125707	Proteosome
miR-99b-5p; miR-375-3p; miR-484; miR-370-3p; miR-99a-5p; miR-574-3p; miR-532-3p; miR-92a-3p; miR-100-5p; miR-197-3p; miR-331-3p; miR-425-5p; miR-122-5p; miR-320a-3p; miR-433-3p; miR-191-5p; miR-200c-3p; miR-483-5p; miR-200a-3p; miR-134-5p; miR-193b-5p; miR-28-3p; miR-492; miR-339-3p; miR-328-3p; miR-222-3p; miR-885-5p; miR-539-5p	0.000684	0.0125707	Thyroid hormone signaling pathway
miR-375-3p; miR-484; miR-370-3p; miR-99a-5p; miR-574-3p; miR-532-3p; miR-92a-3p; miR-100-5p; miR-197-3p; miR-331-3p; miR-425-5p; miR-122-5p; miR-320a-3p; miR-433-3p; miR-191-5p; miR-200c-3p; miR-483-5p; miR-200a-3p; miR-146b-3p; miR-134-5p; miR-193b-5p; miR-28-3p; miR-492; miR-339-3p; miR-328-3p; miR-222-3p; miR-885-5p; miR-539-5p	0.000704	0.0125707	Neurotrophin signaling pathway
miR-375-3p; miR-484; miR-370-3p; miR-99a-5p; miR-574-3p; miR-92a-3p; miR-100-5p; miR-197-3p; miR-331-3p; miR-425-5p; miR-122-5p; miR-320a-3p; miR-433-3p; miR-200c-3p; miR-483-5p; miR-200a-3p; miR-146b-3p; miR-193b-5p; miR-492; miR-339-3p; miR-328-3p; miR-222-3p; miR-885-5p	0.000719	0.0125707	Toll-like receptor signaling pathway, possibly involved in inflammation
miR-484; miR-99a-5p; miR-532-3p; miR-92a-3p; miR-100-5p; miR-197-3p; miR-331-3p; miR-425-5p; miR-122-5p; miR-320a-3p; miR-191-5p; miR-483-5p; miR-146b-3p; miR-193b-5p; miR-222-3p	0.000967	0.0125953	Aminoacyl-tRNA biosynthesis
